# Smoking and Inflammation

**DOI:** 10.1371/journal.pmed.0020198

**Published:** 2005-06-28

Smoking is the single largest preventable cause of disease and premature death, according to the World Health Organization. Smoking-related diseases kill one in ten adults globally, i.e., 4 million deaths annually; by 2030, if current trends continue, smoking will kill one in six people. Smoking is a prime factor in heart disease, stroke, and chronic lung disease, which cost the United States more than $150 billion a year. The relationship between smoking and cardiovascular disease is well documented, as is the association of smoking with increased levels of inflammatory markers and accelerated atherosclerosis. It is also well known that when smokers quit, their risk of mortality and future cardiac events declines, but there is little data quantifying the rate of this risk reduction.

Smoking triggers an immunologic response to vascular injury, which is associated with increased levels of inflammatory markers, such as C-reactive protein and white blood cell count. Several studies have shown that such markers predict future cardiovascular events. Markers such as C-reactive protein are also increasingly implicated in the pathogenesis of atherosclerosis. There are, however, still some gaps in our knowledge of cardiovascular disease, smoking, and the predictive use of such markers. For example, few studies have examined the impact of smoking cessation on levels of inflammatory markers or on cardiovascular risk reduction; the level and rate at which the inflammatory response subsides following smoking cessation is also uncertain. Furthermore, whether traditional risk factors can explain the decline in cardiovascular risk following smoking cessation is also unclear. [Fig pmed-0020198-g001]

**Figure pmed-0020198-g001:**
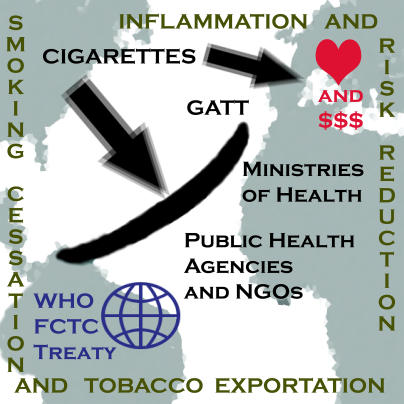
Short- and long-term health-care savings may be realized if smoking cessation is made a priority

In this month's *PLoS Medicine*, Arvind Bakhru and Thomas Erlinger investigate the association between smoking and smoking cessation and levels of inflammatory markers and cardiovascular risk factors. Data were gathered on 15,489 US adults between 1988 and 1994 in the Third National Health and Nutrition Examination Survey. Of these, 7,665 were classified as never smokers, 3,459 were former smokers, and 4,365 were current smokers.

The investigators focused on changes in C-reactive protein, white blood cell count, albumin, and fibrinogen, and the traditional risk factors—total cholesterol, high-density lipoprotein cholesterol, triglycerides, systolic blood pressure, and diabetes—that occurred with decreased smoking intensity and increased time since smoking cessation. They found that inflammatory markers had a dose-dependent and temporal relationship to smoking and smoking cessation. They noted that both inflammatory and traditional risk factors improved with less smoking, but as the time since smokers quit increased, inflammatory markers resolved more slowly than traditional cardiovascular risk factors. Still, the smoking-associated inflammatory response returned to normal within five years after smokers quit, suggesting that the vascular effects were reversible and that cardiovascular risk subsides gradually with reduced exposure.

The authors conclude that these findings support the hypothesis that cardiovascular risk falls as inflammatory response falls, and that inflammatory markers are good indicators of this risk reduction. Despite limitations of the study, including possible errors from self-reporting and lack of data on second-hand smoke and newer measures such as interleukin-6 and high-sensitivity C-reactive protein, the inflammatory markers studied here demonstrated a much clearer trend and longer-lasting effect after smoking cessation than traditional risk factors, and hence were more useful and accurate markers of disease.

As with related studies, these results suggest that smoking cessation should be a more prominent goal of public policy, and the authors conclude that policymakers must pursue smoking cessation plans as an opportunity to make savings on health care through cardiovascular risk reduction. Further research should explore the acute phase response in the months after smoking cessation, which this and other studies have not been able to study adequately.

